# Investigation of Presumptive HIV Transmission Associated with Hospitalization Using Nucleotide Sequence Analysis — New York, 2017

**DOI:** 10.15585/mmwr.mm6910a2

**Published:** 2020-03-13

**Authors:** Bridget J. Anderson, Ernest Clement, Randall Collura, Abigail Gallucci, Emily Westheimer, Sarah Braunstein, Karen Southwick, Eleanor Adams, Emily Lutterloh, Charles Gonzalez, Robert McDonald, Hongwei Jia, William M. Switzer, Priti R. Patel, M. Patricia Joyce, Alexandra M. Oster

**Affiliations:** ^1^Bureau of HIV/AIDS Epidemiology, New York State Department of Health; ^2^Bureau of Healthcare Associated Infections, New York State Department of Health; ^3^HIV Epidemiology and Field Services Program, New York City Department of Health and Mental Hygiene; ^4^University at Albany School of Public Health, State University of New York; ^5^Office of the Medical Director, AIDS Institute, New York State Department of Health; ^6^Epidemic Intelligence Service, CDC; ^7^Division of HIV/AIDS Prevention, National Center for HIV/AIDS, Viral Hepatitis, STD, and TB Prevention, CDC; ^8^Division of Healthcare Quality Promotion, National Center for Emerging and Zoonotic Infectious Diseases, CDC.

Since implementation of Standard Precautions* for the prevention of bloodborne pathogen transmission in 1985, health care–associated transmission of human immunodeficiency virus (HIV) in the United States has been rare (*1*). In October 2017, the New York City Department of Health and Mental Hygiene (NYCDOHMH) and the New York State Department of Health (NYSDOH) were notified by a clinician of a diagnosis of acute HIV infection in a young adult male (patient A) without recognized risk factors (i.e., he was monogamous, had an HIV-negative partner, and had no injection drug use) who had recently been hospitalized for a chronic medical condition. The low risk coupled with the recent hospitalization and medical procedures prompted NYSDOH, NYCDOHMH, and CDC to investigate this case as possible health care–associated transmission of HIV. Among persons with known HIV infection who had hospitalization dates overlapping those of patient A, one person (patient B) had an HIV strain highly similar to patient A’s strain by nucleotide sequence analysis. The sequence relatedness, combined with other investigation findings, indicated a likely health care–associated transmission. Nucleotide sequence analysis, which is increasingly used for detecting HIV clusters (i.e., persons with closely related HIV strains) and to inform public health response (*2*,*3*), might also be used to identify possible health care–associated transmission of HIV to someone with health care exposure and no known HIV risk factors (*4*).

## Investigation and Results

**FIGURE 1:**
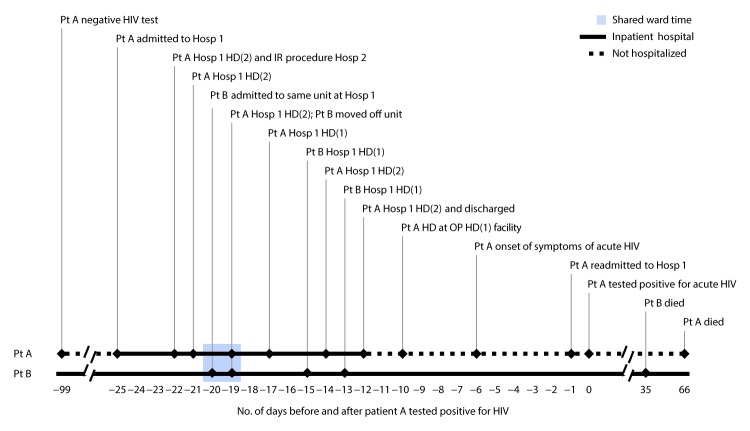
Timeline of key events and potential exposures for patients A and B, with likely health care–associated transmission* of human immunodeficiency virus (HIV) — New York, 2017 **Abbreviations:** HD(1) = hemodialysis machine 1; HD(2) = hemodialysis machine 2; Hosp = hospital; IR = interventional radiology; OP = outpatient; Pt A = patient A; Pt B = patient B. * Estimated from the period during which acute HIV infection can be detected.

Medical record review and interview of patient A by NYCDOHMH and NYSDOH revealed a low risk for HIV acquisition (i.e., monogamous sex with an HIV-negative female partner and no injection drug use). In July 2017, upon admission to hospital 1 for complications of chronic kidney disease (99 days before diagnosis of HIV), patient A’s HIV antigen/antibody rapid test was negative (Figure 1). In October 2017 (25 days before HIV diagnosis), patient A was readmitted to hospital 1 and started hemodialysis. During this admission, patient A underwent vascular access placement by outpatient interventional radiology at hospital 2, and hemodialysis was begun at hospital 1 the same day (22 days before diagnosis). Patient A was discharged 10 days later (12 days before diagnosis) and began hemodialysis at an outpatient dialysis facility 2 days later (10 days before diagnosis).

Patient A was readmitted to the same hospital 9 days later with a 5-day history of fever, sore throat, nausea, vomiting, and diarrhea. The next day, a diagnosis of acute HIV infection was laboratory-confirmed (i.e., HIV-1 and HIV-2 antibody plus HIV-1 p24 antigen test, a negative HIV-1/2 differentiation antibody test, and a detectable HIV-1 RNA qualitative test). The finding of detectable antigen and HIV-1 virus without detectable antibody indicated acute HIV infection (*5*,*6*) and suggested that infection likely occurred 10–22 days earlier, coinciding with the period from his admission to hospital 1 (day −22) (Figure 1) to beginning outpatient hemodialysis (day −10) (Figure 1). Patient A was referred to HIV care but was not prescribed antiretroviral treatment (ART). Sixty-six days after the HIV diagnosis, patient A died from complications related to chronic kidney disease (Figure 1).

Given the likely period when infection occurred was during patient A’s hospitalization, NYSDOH initiated an infection control investigation. A total of 232 patients were identified who had undergone treatment at the same time as patient A on either the hospital 1 inpatient ward or in the hemodialysis unit, or the hospital 2 interventional radiology unit, or the outpatient hemodialysis unit. Using all of the person-identifying information provided by the facilities (i.e., first name, last name, and date of birth) and matching that information against the statewide NYSDOH HIV registry, of the 232, investigators identified 10 persons with previously diagnosed HIV infection. Three were inpatients on the hospital 1 ward with at least one coincident day with patient A’s admission, five received outpatient hemodialysis in the same outpatient hemodialysis unit as patient A, and two received inpatient hemodialysis at hospital 1. Nine of the 10 had documented sustained HIV viral suppression with all viral load results <200 HIV RNA copies/mL throughout 2017. One person with HIV infection diagnosed decades earlier (patient B) was identified as having an increasing HIV viral load between spring and fall 2017. Patient B’s HIV diagnosis was known to hospital 1, and patient B received antiretroviral drugs while in hospital 1. Patient B subsequently died in November 2017. Comparison with the HIV registry of a list of direct care staff members from 1) the hospital 1 ward and hemodialysis unit, 2) the hospital 2 interventional radiology unit, and 3) the outpatient hemodialysis unit yielded no matches.

HIV-1 polymerase (*pol*) sequences generated through standard HIV drug resistance testing and reported as part of HIV surveillance were analyzed by NYSDOH to identify molecular relatedness (*2*,*3*). Patient A and eight of the 10 matched persons, including patient B, had at least one HIV *pol* sequence reported to NYSDOH. Patient A had a November 2017 sequence, and patient B had *pol* sequences available from 2006 and 2010. CDC also generated HIV *pol* sequences from remnant specimens from patients A and B collected in 2017 less than 30 days apart (*6*,*7*).

**FIGURE 2:**
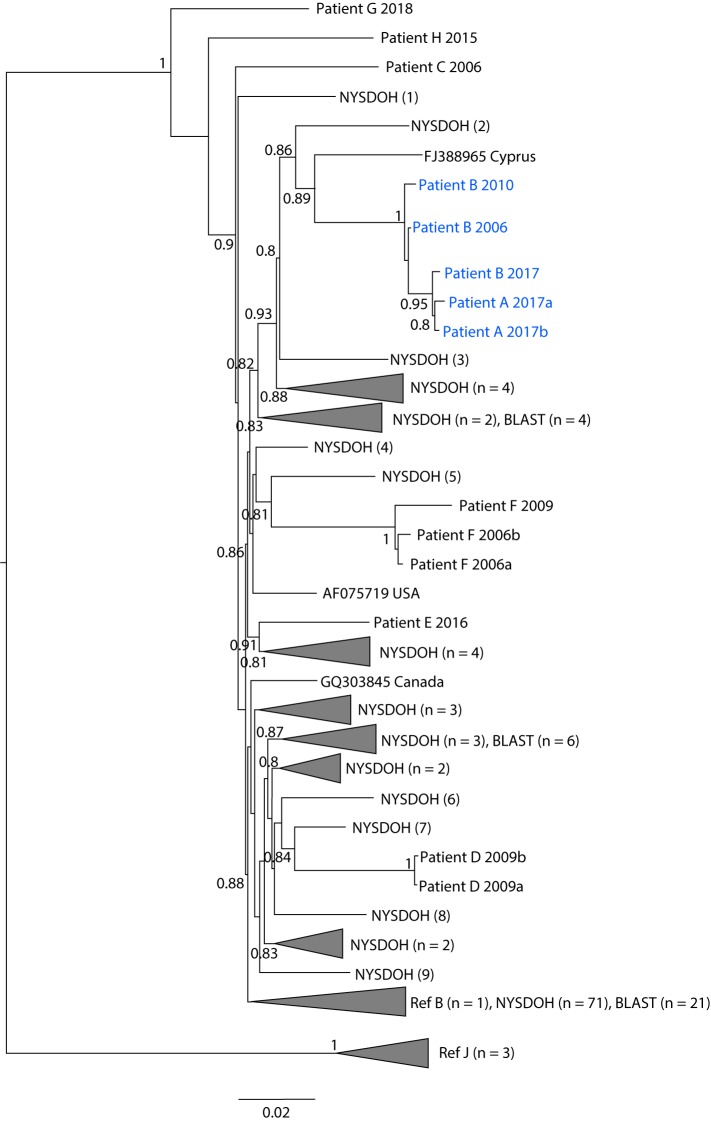
Maximum likelihood phylogeny* of HIV polymerase sequences from patients A and B compared with sequences from other patients and persons in the NYSDOH, CDC, and public databases **Abbreviations:** BLAST = Basic Local Alignment Search Tool; HIV = human immunodeficiency virus; NYSDOH = New York State Department of Health. * Branch length is related to the number of nucleotide substitutions. The more substitutions a sequence has, the longer its branch will be. More evolved sequences will be further from their ancestor. Some nodes are collapsed for better visualization of the whole tree and are depicted as triangles with the number of taxa shown in parentheses. GenBank accession numbers for sequences identified by BLAST analysis are provided when nodes are not collapsed. Confidence values for the branching pattern were assessed with the Shimodaira-Hasegawa (SH) test and are given as probabilities to the left of each branching node. SH values <0.8 are not shown.

All *pol* sequences from patients A and B showed >98% nucleotide identity, with the patient A and patient B sequences from 2017 sharing over 99% identity. HIV-1 sequences for the other nine patients were not closely related to those from patients A or B, showing <96% nucleotide identity (Figure 2). HIV-1 sequences from samples collected from patients A and B were not closely genetically related to those of the 295,000 NYSDOH sequences, 400,000 *pol* sequences available to CDC, or 800,000 HIV sequences at GenBank (as of May 9, 2019). Phylogenetic analysis of the 15 sequences from all 11 patients using maximum likelihood methods showed that all five sequences from patients A and B clustered together strongly in a monophyletic clade with high confidence and with the three 2017 sequences also forming a tight subcluster with high confidence (Figure 2) (*7*).

Medical record review established that, in October 2017, patients A and B were on the same inpatient ward of hospital 1 for 25 hours (shared ward time) (Figure 1). Patients A and B also received inpatient hemodialysis in the same hemodialysis unit. However, they never received hemodialysis on the same day, nor did patient A follow patient B on the same hemodialysis machine. Patient B had no interventional radiology procedures during the hospitalization.

NYSDOH conducted site visits focused on infection control practices at hospital 1’s inpatient ward and hemodialysis unit, hospital 2’s interventional radiology unit, and the outpatient hemodialysis unit. Observations made on hospital 1’s inpatient ward and hemodialysis unit and hospital 2’s interventional radiology unit did not identify any directly observed infection control lapses, nor were opportunities for transmission identified in the hemodialysis unit or interventional radiology unit. The site visits included interviews with clinical providers and other key personnel. Hospital 1 pharmacy records indicated the only medications prescribed to both patients were intravenous saline flushes and injectable darbepoetin. Patient A did not receive narcotics on the hospital 1 ward. Hospital 1 used 3 mL and 10 mL prefilled, plastic-wrapped, sealed, saline syringes stored in locked clean utility rooms. Darbepoetin (used to treat anemia related to chronic kidney disease) was supplied in patient-specific, prefilled, single-use syringes of various strengths delivered by the hospital A pharmacy to the hemodialysis unit. All other medications were tracked and dispensed via a biometric-controlled and password-controlled automated dispensing system.

Patients A and B had no known social contact, and no specific mechanism for transmission between these patients was confirmed. However, the epidemiologic evidence and high degree of viral genetic relatedness were most compatible with transmission having occurred at hospital 1 during mid-October 2017 (Figure 1).

## Public Health Response

NYSDOH recommended a notification of potential exposure to bloodborne pathogens at hospital 1 for any patient who had an injection, infusion, or other invasive procedure while an inpatient on the same unit in hospital 1 or who received inpatient hemodialysis at hospital 1 during the period when both patients A and B were inpatients at hospital 1 (days −20 to −12 before patient A tested positive for HIV) (Figure 1). The hospital mailed letters to the 36 living patients meeting NYSDOH criteria; the letters described potential HIV exposure and offered free testing for HIV as well as for hepatitis B and hepatitis C viruses, although neither patient had hepatitis B or hepatitis C infections. Ongoing surveillance has not identified any additional cases related to this investigation.

## Discussion

In this investigation of acute HIV infection with a narrow transmission window, low reported behavioral risks associated with HIV acquisition and the timing and results of HIV testing indicate the infection likely occurred when patient A was hospitalized. Analysis of HIV nucleotide sequence data for persons with overlapping health care exposures helped to identify a possible source of infection.

The inpatient hemodialysis unit, interventional radiology unit, and outpatient hemodialysis unit were excluded as likely transmission locations because of an absence of a source patient or opportunity for transmission. Although no specific infection control lapses were directly observed, the epidemiologic data and nucleotide sequence analyses provide support for possible health care–associated transmission while both patients were hospital 1 inpatients on the same ward. However, the possibility cannot be excluded that transmission involved a person (hospitalized or not) with undiagnosed HIV infection or a person with diagnosed HIV infection without an available HIV-1 *pol* sequence for comparison.

This incident serves as a reminder of the importance of strict adherence to Standard Precautions within health care settings. It also underscores the utility of sequence analysis to identify transmission to persons with no known HIV risk factors through uncommon health care routes that might otherwise go unrecognized.

**Summary**

**What is already known about this topic?**

Health care–associated human immunodeficiency virus (HIV) transmission is uncommon in the United States. Adherence to Standard Precautions can help to prevent health care–related spread of bloodborne pathogens.

**What is added by this report?**

In this investigation of an acute HIV infection in a patient with chronic kidney disease who received care in a hospital and other health care settings, epidemiologic and nucleotide sequence data support likely health care–associated transmission.

**What are the implications for public health practice?**

Investigators of acute HIV infection in persons with recent health care exposure and no known risk factors for HIV might consider the possibility of health care–associated transmission and conduct nucleotide sequence analysis.

## Footnotes

* https://www.cdc.gov/infectioncontrol/basics/standard-precautions.html.
